# Efficacy of sulfadoxine-pyrimethamine in Tanzania after two years as first-line drug for uncomplicated malaria: assessment protocol and implication for treatment policy strategies

**DOI:** 10.1186/1475-2875-4-55

**Published:** 2005-11-18

**Authors:** Kefas Mugittu, Salim Abdulla, Nicole Falk, Honorati Masanja, Ingrid Felger, Hassan Mshinda, Hans-Peter Beck, Blaise Genton

**Affiliations:** 1 Ifakara Health Research and Development Centre (IHRDC), P. O. Box 53, Ifakara, Tanzania; 2 Swiss Tropical Institute, Socinstrasse 57, 4002 Basel, Switzerland

## Abstract

**Background:**

Systematic surveillance for resistant malaria shows high level of resistance of *Plasmodium falciparum *to sulfadoxine-pyrimethamine (SP) across eastern and southern parts of Africa. This study assessed *in vivo *SP efficacy after two years of use as an interim first-line drug in Tanzania, and determined the rates of treatment failures obtained after 14 and 28 days of follow-up.

**Methods:**

The study was conducted in the Ipinda, Mlimba and Mkuranga health facilities in Tanzania. Children aged 6–59 months presenting with raised temperature associated exclusively with *P. falciparum *(1,000–100,000 parasites per μl) were treated with standard dose of SP. Treatment responses were classified according to the World Health Organization (WHO) definition as Adequate Clinical and Parasitological Response (ACPR), Early Treatment Failure (ETF), Late Clinical Failure (LCF) and Late Parasitological Failure (LPF) on day 14 and day 28.

**Results:**

Overall 196 (85.2%) of 230 patients had ACPR on day 14 but only 116 (50.9%) on day 28 (57.7% after excluding new infections by parasite genotyping). Altogether 21 (9.1%) and 13 (5.7%) of the 230 patients assessed up to day 14 and 39 (17.1%) and 55 (24.1%) of the 228 followed up to day 28 had clinical and parasitological failure, respectively.

**Conclusion:**

These findings indicate that SP has low therapeutic value in Tanzania. The recommendation of changing first line treatment to artemether + lumefantrine combination therapy from early next year is, therefore, highly justified. These findings further stress that, for long half-life drugs such as SP, establishment of cut-off points for policy change in high transmission areas should consider both clinical and parasitological responses beyond day 14.

## Background

There is controversy over the therapeutic life of sulfadoxine-pyrimethamine (SP) when used alone for the treatment of uncomplicated malaria in Africa. Experts do not all agree on which drug efficacy measurements more accurately predict usefulness of a drug in a community. Some consider clearance of symptoms alone [1] or plus parasites by day 14, as advised by the World Health Organization [Regional Office for Africa (WHO/AFRO) Consultative Meeting on Antimalarial Policy in the Africa Region, 14^th^–15^th ^August 2003, Harare, Zimbabwe]. Others regard clearance of both symptoms and parasites over a much longer period as the most accurate measure of drug effectiveness [2-4].

The assessment methodology has profound implications in terms of treatment policy strategies. Attempts have been made to define the cut-off points for changing first-line malaria treatment. Using the old treatment response classification criteria, the action period was due when a combined Early Treatment Failure (ETF) and Late Treatment Failure (LTF) were between 16 – 24% [4,5]. A couple of years ago, WHO/AFRO recommended 15% clinical and 25% parasitological treatment failure rates at day 14 as cut-off points for implementation of policy change in intense transmission areas (WHO/AFRO consultative meeting on antimalarial policy in the Africa region, 14^th^–15^th ^August 2003, Harare, Zimbabwe).

Systematic surveillance for efficacy of antimalarial drugs shows increasing levels of *Plasmodium falciparum *resistance to SP across eastern and southern parts of Africa [6,7]. In 2001, Tanzania adopted SP as interim first-line treatment for uncomplicated malaria while awaiting for the results of different combination therapies trials. As part of the National Malaria Control Programme (NMCP), this study assessed *in vivo *SP efficacy after two years of widespread use in Tanzania.

## Methodology

The study was conducted from July to November 2003 in the Ipinda (south-west), Mlimba (south-east) and Mkuranga (east) health facilities in Tanzania. Malaria transmission in these areas is perennial with peaks between May and July. A slightly modified WHO antimalarial drug efficacy testing protocol [8] was used, so as to conform with another study that was being conducted at the same time under the same project framework in Papua New Guinea, in areas with lower levels of endemicity. Children aged 6–59 months presenting with raised temperature (37.5°C–39.5°C) associated with *P. falciparum *parasitaemia between 1,000–100,000 parasites per μl were recruited. Exclusion criteria and other procedures were as detailed in the protocol [8]. Patients were treated (under observation) with a standard dose of SP (Fansidar^® ^Roche), i.e 1.25 mg/kg of pyrimethamine and 25 mg/kg of sulfadoxine. The responses were classified according to the new WHO definition as ACPR, ETF, LCF and LPF at day 14 and day 28 [8].

Treatment failures rates were corrected after genotyping the *msp2 *locus to detect new infections. Extensive diversity in this locus has been observed with over 84 allelic variants in south-eastern Tanzania [9,10] and other investigators observed high genotype complexity with an average of 4.9 genotypes per asymptomatic individual in eastern Tanzania [11]. These observations are suggestive that *msp2 *alone may sufficiently discriminate recrudescence from reinfection in Tanzania. It has previously been shown that analysis of *msp2 *locus alone can effectively distinguish recrudescence from reinfection in Uganda [12]. The clinical and molecular data were combined and analysed using Stata version 8.0 (Stata Corporation Inc, Texas, USA).

## Results

A total of 241 patients were recruited, of which 13 were lost to follow-up. Table 1 summarizes patient age and clinical parameters recorded on admission day by site. Table 2 provides details of treatment outcome by site. On day 28, only 116 (50.9%) of the 228 patients showed ACPR. Molecular genotyping showed that 27/112 (24.5%) recurrent infections were due to re-infections, therefore were excluded from the analysis and recorded as withdrawn. Hence, PCR-corrected ACPR was 116/201 (57.7%). 196 (85.2%) of the 230 patients had ACPR by day 14.

**Table 1:** Mean age, temperature, haemoglobin and parasite density on admission day

**Site**	**Means**				
	**Weight in kg(SD)^1^**	**Age in years (SD)^1^**	**Temperature in °C (SD)^1^**	**Hb^1 ^in g/dl (SD)^1^**	**parasites/μl (SD)**

Ipinda (n = 73)	10.7(2.9)	1.7(1.3)	38.4(1.0)	9.4(1.6)	31'499 (30,260)
Mlimba (n = 75)	11.0(2.9)	1.7(1.4)	38.3(0.9)	9.0(1.6)	53,206(30,782)
Mkuranga (n = 93)	10.9(3.7)	1.3(1.0)	38.7(0.8)	8.6(1.9)	44,877(38,572)

Hb = haemoglobin; SD = standard deviation

**Table 2:** Sulfadoxine-pyrimethamine treatment outcomes

**Results**	**no. of included patients**	**LF and corr.**	**Evaluable patients**	**ACPR ** **n (%)**	**ETF** **n (%)**	**LCF** **n (%)**	**Total CF** **n (%)**	**LPF** **n (%)**	**Overall TF** **n (%)**
**At D14**									
Ipinda	73	1	72	62(86.1)	7(9.7)	2(2.8)	9(12.5)	1(1.4)	10(13.9)
Mlimba	75	3	72	63(87.5)	3(4.3)	4(5.7)	7(9.1)	2(2.9)	9(12.9)
Mkuranga	93	7	86	71(82.6)	3(3.5)	2(2.3)	5(6.9)	10(11.6)	15(17.4)
Total	241	11	230	196(85.2)	13(5.7)	8(3.5)	21(9.1)	13(5.7)	34(14.9)
**At D28**									
Ipinda	72	0	72	44(61.1)	7(9.7)	5(6.9)	12(16.7)	16(22.2)	28(38.9)
Mlimba	72	2	70	34(48.6)	3(4.3)	9(12.9)	12(17.1)	24(34.3)	36(51.4)
Mkuranga	86	0	86	38(44.2)	3(3.5)	12(13.9)	15(17.4)	33(38.4)	48(55.8)
Total	230	2	228	116(50.9)	13(5.7)	26(11.4)	39(17.1)	55(24.1)	112(49.1)
**At D28 PCR-corrected**									
Ipinda	72	6	66	44(66.7)	7(9.7)	5(6.9)	12(16.7)	10(13.9)	22(33.3)
Mlimba	70	10	60	34(56.7)	3(4.3)	5(7.1)	8(11.4)	18(25.7)	26(43.3)
Mkuranga	86	11	75	38(50.7)	3(3.5)	8(9.3)	11(12.8)	26(30.2)	37(49.3)
Total	228	27	201	116(57.7)	13(5.7)	18(7.9)	31(13.6)	54(23.7)	85(42.3)

ACPR = Adequate Clinical and Parasitological Response; TF = Treatment failure, ETF = Early Treatment Failure, LCF = Late Clinical Failure; LPF = Late Parasitological Failure; CF = Clinical failure, n = Sample Size; TF = Treatment failure, FP = Lost to Follow-up, corr. = corrected

The total clinical failure by day 14 and 28 was observed in 21 (9.1%) out of 230 and 39 (17.1%) out of 228 patients, respectively. 13 (5.7%) and 55 (24.1%) of the patients had LPF by day 14 and 28, respectively. Thus 34 (14.9%) out of 230 and 112 (49.1%) out of 228 patients had overall treatment failure by day 14 and 28, respectively. After genotyping recurrent infections day-28 treatment failures decreased to 85 (42.3%). In this study Mkuranga recorded the highest rates of both PCR-adjusted and unadjusted treatment failures followed by Mlimba and Ipinda.

## Discussion

In 2001 Tanzania replaced chloroquine with SP as interim first-line antimalarial drug. Prior to this change, baseline clinical trials with SP had been conducted throughout the country using the 14 day follow-up protocol, and indicated an average efficacy of 86% on day 14. These findings paved the way for the malaria treatment policy change [13] and SP is still used as first line antimalarial drug in Tanzania. The present study assessed SP efficacy (using a 28 day follow-up) in three sites in Tanzania after two years of use as first-line antimalarial drug. With this extended period of follow-up, half of the patients (49.1%) failed treatment. Even when new infections were taken into account by genotyping, the overall treatment failure rate (42.3%) was still high. This level of resistance is close to that observed in Muheza (45%), an area of high SP resistance in Tanzania [14].

Restricting our analysis to outcomes at day 14 would have led to misleadingly low clinical (9.1%) and parasitological (5.7%) treatment failure rates with the overall treatment failure (14.9%) being equal to that recorded at baseline prior to policy change. Using a shorter follow-up period, another study in parts of Tanzania also recorded an overall SP treatment failure of only 9.2% [15]. According to WHO/AFRO proposed thresholds for policy change (i.e 15% and 25%, respectively), these failure levels would still be considered acceptable. Retention of SP clinical efficacy at day 14 after 10 years of its use as first-line drug has been demonstrated in Malawi [1]. However, extending the follow-up to day 28 the total failure was as high as 66%. Even at day 14 the clinical and total failure rates were far above 15% and 25%, respectively. As in Malawi, the majority of the recurrent infections in our study were LPFs observed between day 14 and 28. The new WHO efficacy testing protocol [8] recommends follow up for 28 days for drug with long half-life such as SP, if genotyping can be done to distinguish recrudescence from re-infections. When efficacy assessment is based only on clearance of symptoms in the first 14 days, the level of parasite resistance can be grossly underestimated. It is widely accepted that clearance of both parasitaemia and symptoms is the most accurate measure of the intrinsic resistance of the parasite to a drug [2-4]. The resistant parasite that is apparently causing asymptomatic infection in LPF is likely to lead in the short-term to a new clinical episode [14] and/or to anaemia, depending on the immunity of the subject.

Our observations show that SP efficacy in Tanzania is compromised and fully justify the recent decision to review the current malaria treatment policy from early next year in favour of artemether + lumefantrine combination therapy. This recommendation should be implemented at a large scale as soon as possible. Such a change would be welcome to protect the use of SP in its indication for the intermittent preventive treatment of pregnant women (IPTp). Indeed, it is at present the only drug that can be used for IPTp purpose because of its good safety profile. As far as methodology is concerned, the findings stress that cut-off points for malaria treatment policy change in high transmission areas should consider both clinical and parasitological responses beyond day 14, coupled with distinction of recrudescence from re-infection using molecular genotyping.

## Authors' contributions

B. Genton, H-P. Beck and K. Mugittu designed the study. S. Abdulla and K. Mugittu organised the clinical work. H. Mshinda was the supervisor in Tanzania. N. Falk, I. Felger, H-P. Beck performed molecular genotyping. H. Masanja carried out the statistical analysis. K. Mugittu and B. Genton wrote the article and all others contributed to it.

## Ethical approval

The study was approved by the Tanzanian national and institutional ethics bodies
